# Brain on FIRES: Super Refractory Seizure in a 7 yr Old Boy

**Published:** 2016

**Authors:** Alireza Tavasoli, Behdad GHARIB, Houman ALIZADEH, Hossein FARSHADMOGHADDAM, Sara MEMARIAN, Mahmoodreza ASHRAFI, Meisam SHARIFZADE

**Affiliations:** 1 Pediatric Neurology Department, Children’s Medical Center, Tehran University of Medical Sciences, Tehran, Iran; 2 Pediatric Intensive Care Department, Children’s Medical Center, Tehran University of Medical Sciences, Tehran, Iran; 3 Radiology Department, Children’s Medical Center, Tehran University of Medical Sciences, Tehran, Iran; 4 Pediatrics Department, Children’s Medical Center, Tehran University of Medical Science, Tehran, Iran

**Keywords:** Refractory seizure, Infectious encephalitis, Lateralized seizure, Autoimmune encephalitis

## Abstract

We present a 7 yr old boy afflicted with super-refractory seizure that responded poorly to antiepileptic drugs and sustained a long course of hospitalization and complications of high doses of medications as well as longstanding stay in hospital. The differential diagnoses were, fever-induced refractory epileptic encephalopathy (FIRES), and infectious and autoimmune encephalitis. However, work-ups had not revealed any evidence of any specific diagnosis, so we assumed that he was afflicted by viral infectious encephalitis as he had, fever, vomiting, and prodromal symptoms of infectious (most probably viral) disease prior to onset of the seizure attacks.

## Case presentation

A 7 yr old boy was referred to our hospital (Children’s Medical Center, Tehran, Iran) with the history of ‘fever’, ‘pharyngitis’, and ‘reduced level of consciousness’ (LOC). Five days prior to the initiation of the impaired level of consciousness and lethargy, he had had fever, sore throat, headache, lymphadenopathy of back of the neck, skin exanthema, pharyngitis and post nasal drip and was administered, benzathin penicillin and cefuroxime. 

The day prior to hospitalization, he felt lethargic and generalized weakness, and developed status epilepticus with mixed type seizure (partial seizure presented by corner of lip twitching and eye blinking and myoclonic jerks of left upper and lower limbs) and as the seizure was not controlled with phenytoin, phenobarbital and midazolam continuous infusion, doctors of another hospital decided to induce barbiturate coma with pentobarbital. He was also prescribed the following medications: vancomycin, meropenem, acyclovir, azithromycin, ribavirin, oseltamivir and dexamethasone, with the impression of infectious encephalitis, however no germ was recognized in the blood and cerebrospinal fluid cultures, and three lumbar punctures performed later in the evolution of the illness were normal. His past medical history was unremarkable except for having contact with travelers to Haj (a Muslims’ pilgrimage ceremony, where Muslims gather around from all over the world in Saudi Arabia, and occasionally cases of meningitis, and new species of influenza have been reported among the travelers).

We received the patient on the third day of LOC with Glasgow coma scale (GCS) of 6 without spontaneous respiration and attached to the mechanical ventilator. On physical examination, his weight was 23 kg, no deep tendon reflex was detected, oculocephalic reflex was normal, Babinski reflex was unresponsive, heart and pulmonary auscultation were normal, the pupils were midsized and non-reactive to light and liver border located 4 centimeters below the costal border, pulse rate= 140 beats per min, and blood pressure= 104/72 cm Hg.

The blood work results were as follows; white blood cells: 1900/μl, neutrophils: 55% (absolute neutrophil count=1045), lymphocyte= 45%, hemoglobin=10.9 gr/deciliter, platelet= 104000/ μl, blood urea nitrogen (BUN)= 11 mg/ deciliter, creatinine= 0.7 mg/ deciliter, lactate dehydrogenase (LDH)= 1047 IU/l (normal range up to 746), AST= 60 U/l (up to 37), ALT= 28 U/l (up to 41), C-reactive protein (CRP)= 7.3 milligram/liter (normal range up to 6), erythrocyte sediment rate= 13 mm/h (normal range up to 10), calcium= 7.7 mg/ deciliter (normal range 8.8-10.2), sodium= 133 mill equivalent/l, potassium= 4 milliequivalent/l and normal serum ammonia and coagulation profile.

The complementary blood and cerebrospinal laboratory test were negative for autoimmune disease (antithyroid peroxidase and anti-double-stranded DNA antibodies), metabolic disease, thyroid diseases, immunodeficiency disorders, and PCR of cat scratch disease, CMV, HSV, HHV 5 and 6, Corona virus, influenza, Ebstein- Barrand JC virus. Later in the course of the disease, the complete panel of CSF for autoimmune encephalitis was also performed, which was negative. The patient’s first diagnosis was super refractory seizure caused by infectious encephalitis and hospitalized for 77 days. 

**Fig 1:**
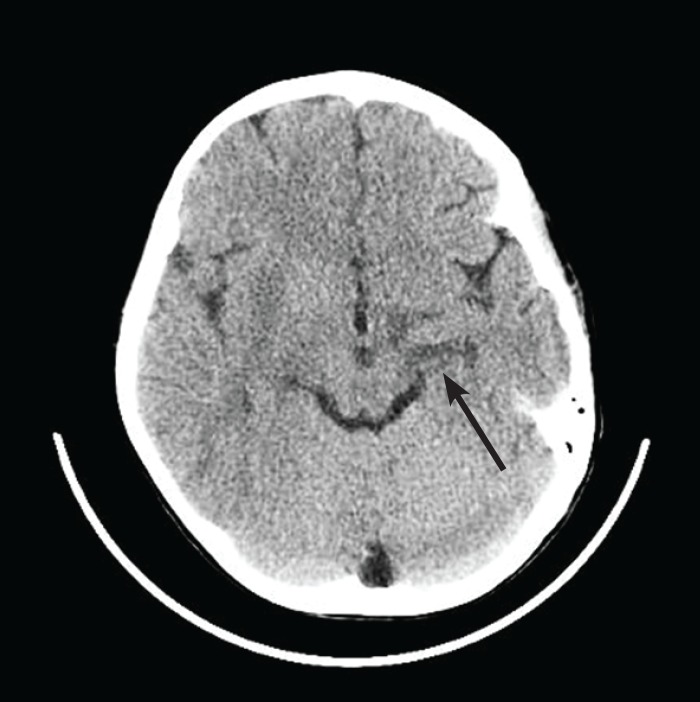
Faint low attenuated areas in the left lentiform nucleus at the level of midbrain are seen

On the 1st day of admission, we started with midazolam and phenobarbital. As the convulsions continued, the neurologist consultant recommended administering repeated loading dose and increasing gradually the maintenance dose to the maximum level of, phenytoin, phenobarbital and sodium valproate. Propofol (load and maintenance) was started, and midazolam infusion increased to 10 mcg/kg/min, but without success in controlling seizure. The seizures started with simultaneous loss of consciousness and seemed to be very sensitive to tactile stimulus from the beginning, and were mostly localized on left side. Because of hypotension caused by side effects of antiepileptic drugs, norepinephrine 0.05 microgram/kg/min started and echocardiography performed and revealed that the ejection fraction of heart was 50-60%. Brain computerized tomography (CT scan) was performed and revealed; faint low attenuated areas in the right Lentiform nucleus at the level of midbrain (Figure 1).

On the 2nd day, mannitol infusion and other measures for cerebral edema (seen on brain computerized tomography) were administered. On the 3rd day, he had myoclonic seizures in the left hand, and sodium valproate continuous infusion, topiramate (25 mg/twice a day per gavage) was started and considered the possibility of autoimmune encephalitis, high dose of methyl prednisolone (500 mg intravenous/d for 5 d) was also administered but did not affect the course of seizures. Because of hypotension, pentobarbital discontinued.

On the 4th day, as the super refractory seizure was not controlled, midazolam discontinued, and pyridoxine (100 mg intravenous daily), chloral hydrate (15 cc/4 h via enteral route from the 1% solution), topiramate (increased to 50 mg twice a day), thiopental, intravenous immunoglobulin (IVIG), and levetiracetam started. The probable diagnoses were considered autoimmune encephalitis or infectious-related epileptic encephalopathy.

Cerebro-spinal fluid (CSF) analysis was normal and was also negative for autoimmune encephalitis. On the 5th day, sodium valproate continuous infusion changed to the intermittent dose (160 mg/ 3 times a day) and diazepam load and continuous infusion started. 

On the 6th day, he developed cerebral salt wasting. On the 7th day, ketamine infusion (14 mg/h) started and continued with gradual increments, still without any success in controlling seizures.

After 2-3 weeks, the seizure attacks were reduced, and with gradual tapering of the drugs, dyskinesia phenomenology compatible with “Dystonic tremor” and chorea appeared. Therefore, we prescribed, baclofen, artan, madopar (levodopa and benserazide) and tetrabenazine, which were partially effective. The dyskinesia continued even during comatose condition. Moreover, the initiation of these movements was also very sensitive to touch and sound. An electroencephalogram (EEG) revealed ‘’generalized multifocal paroxysmal epileptic discharge’’ and because of status dystonicus type of dyskinesia, madopar, clonidine and tetrabenazine were continued which seemed to be effective partially. 

**Fig 2:**
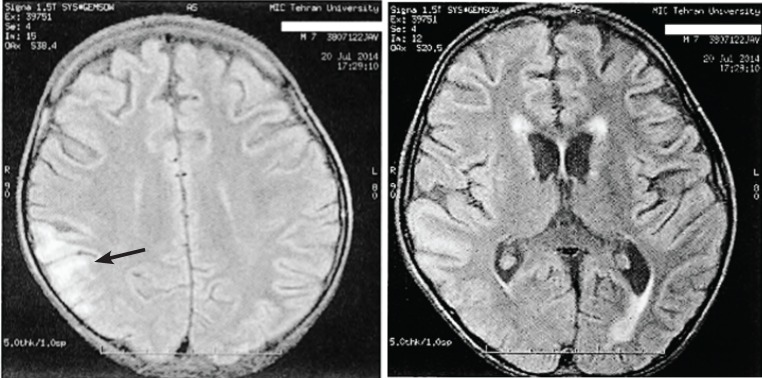
Magnetic resonance imaging (MRI) in FLAIR sequence showing ‘abnormal right parietal corticosubcortical high signal’

The focal convulsion continued with presentation of, ‘’jaw lock, drooling, lateral gaze, dystonia, corner of lip twitching and blinking’’. On the 40th day of hospitalization; chorea and dystonia continued but the pattern of dyskinesia started changing gradually to a movement disorder, as they stopped during sleeping state. Therefore, trihexyphenidyl was added to the medication list. Magnetic resonance imaging (MRI) in FLAIR sequence on the 50th day of admission revealed “abnormal right parietal cortico-subcortical high signal’ (Figure 2).

During his long stay he sustained the problems of prolonged hospitalization such as nosocomial blood stream infection, gastric content pulmonary aspiration, drug induced neutropenia, tracheal tube dislocation and intubation and extubation procedures, tracheostomy, and low blood pressure as the side effect of antiepileptic medications, managed accordingly. He also afflicted by left wrist flexion deformity and spasm, that continued after discharge. 

The long list of antiepileptic medications prescribed (gradually increased and tapered off or continued, according to the useful effects and side effects) were as follows:

Phenytoin (day 1 to 38), phenobarbital (day 1 to ‘discharged with’), pentobarbital (day 1 to 3), sodium valproate (day 1 to ‘discharged with’), midazolam (day 2 to 5, as continuous infusion and intermittent doses), propofol (day 2 to 3), topiramate (day 3 to ‘discharged with’), high dose methylprednisolone (day 3 to 9), IVIG (day 4 to 7), clonazepam (day 4 to 39), pyridoxine (day 4 to ‘discharged with’), levetiracetam (day 4 to ‘discharged with’), thiopental (day 4 to 7),chloral hydrate (day 4 to 10), diazepam (day 4 to 35 as continuous infusion and intermittent doses), vigabatrin (day 5 to 63), ketamine infusion (day 8 to 15), and carbamazepin (day 28 to 52). 

Around the 45th – 50th day of hospitalization the seizure activity reduced significantly, and on the day 77, the patient discharged with; phenobarbital tablet (30mg in morning, 60 mg in night), pyridoxine tablet (60 mg daily), topiramate tablet (25 mg in morning, 50 mg in night), epilim syrup (200 mg/5cc, 5cc * 3 times a day), levetiracetam (500 mg 3 times a day), trihexyphenidyl (2 mg 3 times a day), risperidone tablet (0.5 mg 2 times a day), and carnitine syrup.

On the follow-up visits, he had not had any seizure activity for about 4 months after discharge. The seizure activity started for the first time after discharge, triggered by staying awake till morning and playing computer games, in sleep state, and it was in figure of left upper and lower limb jerk, and left hemifacial twitching, which extended to both lower limb jerk. This episode lasted shortly and a similar episode occurred one month later (5 month after discharge), and since the latter episode, he has had several episodes (2-3 times per month) and all of the seizures were associated with loss of consciousness (predominantly partial type with staring) without loss of muscle tone, and lateralized on the left side. 

One year after discharge, he still has seizure activity with the frequency of about 1-2 times weekly which lasts 1-2 min, as mentioned above. He also has minimal cognitive dysfunction, slurred speech and flexion deformity of left hand. His weight is 37 kg now and is receiving the following list of medications: topiramate 50 mg/twice a day, levetiracetam 500mg/twice a day, phenobarbital 50 mg/ twice a day, valproate sodium 400 mg/twice a day, pregabaline 25 mg/three times a day (for neuropathic pain of the left hand), thiamine 100 mg and pyridoxine 40 mg twice a week.

## Discussion

“When status epilepticus continues or recurs in spite of 24 hours of general anesthesia, it is defined as superrefractory status epilepticus” (8). Encephalopathies or another central nervous (CNS) infection should be considered when the patient has a group of symptoms of recent febrile illness with behavior, cognition, consciousness and personality changes or new focal neurological signs (6). The differential diagnosis of encephalopathy in children includes a wide range of etiologies such as metabolic, genetic, traumatic, infectious, para-infectious, toxic and malignancies (4). Inflammation caused by etiologies such as direct invasion of brain parenchyma, antibodies directed against components of CNS, has an important role in status epilepticus (SE) (2).

FIRES is an infection-related epileptic encephalopathy with undefined etiology. It has many names like, Devastating epileptic encephalopathy in school-aged children (DESC), Acute encephalitis with refractory repetitive partial seizures (AERRPS), Idiopathic catastrophic epileptic encephalopathy, and New onset refractory status epilepticus (NORSE). Severe refractory status epilepticus due to presumed encephalitis. Genetic susceptibility and inflammation have been proposed as the etiologies (3). In FIRES, children develop seizure that rapidly turns into SE. This situation occurs a few days to one week after a non-specific febrile infection. 

The chronic phase continues after the acute phase characterized by pharmaco-resistent epilepsy. Seizures are usually generalized with focal onset. Weeks or months after the initiation of the illness, seizures decrease or stop, and intractable epilepsy starts a few weeks to 3 months after the end of SE (2). The prognosis of FIRES is poor (3).

All patients afflicted by FIRES have an infection one week before the beginning of the symptoms which is mostly respiratory tract infection (5).

Autoimmune encephalitis is a group of disorders associated with antibodies against the surface proteins of neuronal cells and synaptic receptors. They have a wide range of symptoms including seizure, behavioral changes, psychosis, catatonia, autonomic dysregulation and abnormal movements (10). Some patients afflicted by autonomic encephalitis may progress to a more generalized encephalopathy with movement disorder (4). Nonspecific viral infections may lead to breaking immune tolerance and increase the possibility of the antibodies to enter CNS by increasing the permeability of the blood-brain barrier. Occasionally a tumor that produces the target neuronal antigen likely contributes in triggering the immune response. However in many of autoimmune encephalitis disorders the blood brain barrier seems to be intact and the autoantibodies may be synthetized inside the CNS (10).

Perhaps HSV is the most frequent cause of sporadic encephalitis, and have a tendency to affect the temporal lobe (1).

In herpes encephalitis negative result of polymerase chain reaction for HSV, does not rule out the infection and treatment with acyclovir should be completed for 21 days (9). A cerebro-spinal fluid sample without abnormal cells has been described for varicella zoster virus, Epstein- Barr virus and cytomegalovirus and is more prevalent in immune-deficient patients (6). 

Systemic infection with influenza virus can cause seizure provoked by fever and systemic illness, extrapyramidal syndromes, Guillain-Barre syndrome, encephalitis and transverse myelitis. H1N1 can lead to encephalopathy and focal findings and there has been no evidence supporting the benefits of antiviral treatment for neurologic course of the disease. A rare type of acute encephalopathy/ encephalitis associated with influenza, can begin with a fulminant neurologic disease and may be accompanied with any influenza viral serotype. This disease may be associated with a genetic disorder in releasing pro- inflammatory cytokines and hypercytokinemia (1).

Human herpes virus type 6 which is a common viral illness in childhood may invade CNS at the time of primary infection and rarely cause a clinical neurologic disease but long-term latent infection may occur, and in case of immunosuppression, acute limbic encephalitis, probably due to reactivation of the virus can occur (1). 

The patient’s problem presented first with an infectious disease (upper respiratory tract infection) and fever that proceeded to vomiting, decreased level of consciousness and refractory seizure with lateralized features. He responded poorly to antiepileptic drugs and sustained a long course of hospitalization and complications of high doses of medications and longstanding stay in hospital. The neurologist consultant even tried ketamine, which has had promising effects in controlling refractory status epilepticus, when midazolam, propofol and phenobarbital, failed to control seizures (7). The differential diagnosis were as follows: Fever-induced refractory epileptic encephalopathy (FIRES), infectious and autoimmune encephalitis, however work-ups had not revealed any evidence of any specific diagnosis, at last we assume that he was afflicted by viral infectious encephalitis as he had, fever, vomiting, and prodromal symptoms of infectious (most probably viral) disease, prior to onset of the seizure attacks. Autoimmune encephalitis was considered because of the refractory course and abnormal movements occurred in the course of the illness and ruled out as the antibodies’panel of cerebro-spinal fluid (CSF) was negative and no response seen to high dose of methylprednisolone and intravenous immunoglobulin administration. 

**In Conclusion, **we did not find abnormal results in CSF analysis and viral PCR work-ups, but as mentioned above occasionally this may happen and clinically we assume viral encephalitis as the first differential diagnosis of our patient. 

## Authors’ Contribution

AR Tavasoli: Drafting. 

B.Gharib: Drafting, Designing, final approval of the work, Interpretation.

H. Alizadeh: Interpretation

H. Farshad moghaddam: Drafting.

S. Memarian: Drafting, Final approval of the work Mahmoodreza Ashrafi: Designing of the work

M. Sharifzade: Drafting, Final approval of the work

All authors agreed to be accountable for all aspects of the work in ensuring that questions related to the accuracy or integrity of any part of the work are appropriately investigated and resolved.
